# Effects of ionizing radiation on surface properties of current restorative dental materials

**DOI:** 10.1007/s10856-021-06543-5

**Published:** 2021-06-12

**Authors:** Débora Michelle Gonçalves de Amorim, Aretha Heitor Veríssimo, Anne Kaline Claudino Ribeiro, Rodrigo Othávio de Assunção e Souza, Isauremi Vieira de Assunção, Marilia Regalado Galvão Rabelo Caldas, Boniek Castillo Dutra Borges

**Affiliations:** Department of Dentistry, Universidade Federal do Rio Grande do Norte (UFRN), Av. Salgado Filho, 1787, Lagoa Nova, Natal, RN CEP: 59056-000 Brazil

## Abstract

To investigate the impact of radiotherapy on surface properties of restorative dental materials. A conventional resin composite—CRC (Aura Enamel), a bulk-fill resin composite—BFRC (Aura Bulk-fill), a conventional glass ionomer cement—CGIC (Riva self cure), and a resin-modified glass ionomer cement—RMGIC (Riva light cure) were tested. Forty disc-shaped samples from each material (8 mm diameter × 2 mm thickness) (*n* = 10) were produced according to manufacturer directions and then stored in water distilled for 24 h. Surface wettability (water contact angle), Vickers microhardness, and micromorphology through scanning electron microscopy (SEM) before and after exposition to ionizing radiation (60 Gy) were obtained. The data were statistically evaluated using the two-way ANOVA and Tukey posthoc test (*p* < 0.05). Baseline and post-radiation values of contact angles were statistically similar for CRC, BFRC, and RMGIC, whilst post-radiation values of contact angles were statistically lower than baseline ones for CGIC. Exposition to ionizing radiation statistically increased the microhardness of CRC, and statistically decreased the microhardness of CGIC. The surface micromorphology of all materials was changed post-radiation. Exposure to ionizing radiation negatively affected the conventional glass ionomer tested, while did not alter or improved surface properties testing of the resin composites and the resin-modified glass ionomer cement tested.

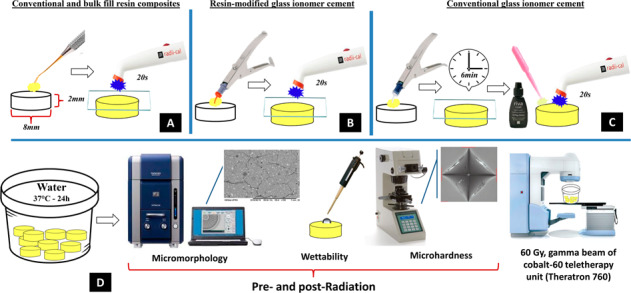

## Introduction

The head and neck region is a prevalent site for the occurrence of malignant neoplasms which mainly affect several oral tissues [1, 2]. The treatment of cancer in the head and neck region is generally based on clinical staging, tumor location, histological differentiation, and the patient’s clinical conditions [3, 4]. Treatment is performed in most situations employing surgery or radiotherapy, and there may be cases that require both association therapy. Radiotherapy uses a beam of ionizing radiation and aims to destroy tumor cells, minimizing damage to normal cells [5, 6].

The management of patients submitted to cervicofacial radiotherapy requires a multidisciplinary approach, making the restoration of caries lesions highly challenging for dentists [7, 8]. The restorative treatment of caries lesions before radiation therapy is necessary to prevent disease progression and reduce the burden of microorganisms [9]. This approach is important because adhesion between restorative materials and dental tissues is compromised by ionizing radiation so that a post-radiotherapy tooth restoration might provide an unsuccessful treatment [10].

Regarding the restorative materials available for dentists, resin composites, and glass ionomer cement are frequently used to restore caries lesions and esthetic restorative treatments [11]. Resin-based restorative dental materials are versatile and have shown constant progress concerning types of filler particles [12]. However, resin composites still present unfavorable aspects such as polymerization shrinkage stress, marginal infiltration, biocompatibility, and the presence of unreacted monomers [13]. Bulk-fill resins emerged to optimize restorative procedures with more advantages, such as the single-step incremental insertion proposal, saving clinical time, demonstrating a better degree of conversion, and polymerization stress in deeper layers [14].

In restorative dentistry, glass ionomer cement is available in two formulations: conventional glass-ionomer and resin-modified glass-ionomer [15]. Conventional glass-ionomers set via an acid-basic reaction and present anti-cariogenic activity, good adhesion to dental tissues, and long-term fluoride release [16]. However, the low tensile strength, susceptibility to dehydration, and low fracture toughness may limit the use [17]. In an attempt to improve the physical properties and minimize risk to moisture, resin-modified ionomer cement has been market, increasing work-time, control over material prey, and improving the hardening process [18, 19].

In this context, studies report that gamma radiation therapy affects the glass ionomers and the resin composite properties [20–22]. It has been demonstrated that gamma radiation increased the microhardness of a glass ionomer cement [23] and can produce free radicals that may improve the microhardness of resin-based materials such as conventional resin composites [24]. However, bulk-fill resin composites were recently introduced in the market so that it is necessary to investigate how the gamma radiation would affect their surface properties in an attempt to provide a safe clinical use in individuals undergoing radiotherapy. Moreover, changes in the wettability of dental restorative materials might increase their susceptibility to microbial adhesion and biofilm formation [25], so that there is the need to investigate if the exposition to gamma radiation would become conventional and bulk-fill resin composites and conventional and resin-modified glass ionomer cements more wettable.

This study aimed to evaluate the influence of ionizing radiation on surface wettability, microhardness, and micromorphology of conventional and bulk-fill resin composites, and conventional and resin-modified glass ionomer cement. The null hypothesis was that ionizing radiation would not alter the surface properties of materials tested.

## Materials and methods

### Experimental design

A factorial design 4 × 2 was developed in this laboratory investigation. The factors under study were: restorative dental materials (conventional resin composite—CRC; bulk-fill resin composite—BFRC; conventional glass ionomer cement—CGIC; and resin-modified glass ionomer cement—RMGIC); and timepoint of analysis (before and after exposition to ionizing radiation). Surface microhardness, wettability, and surface morphology were the response variables. The materials used in this study are shown in Table 1.

**Table 1:** Commercial name, manufacturers, material, chemical composition^a^, batch number of materials used in this study

Commercial name	Material	Composition (wt%)	Batch
Aura Enamel, SDI, Victoria, Australia	Conventional resin composite—CRC	Diurethane dimethacrylate (3–20), triethylene glycol dimethacrylate (0.01–7), 2,2-bis[4-(2-methacryloxy)ethoxyphenyl]propane (15–18)	150743
Aura Bulk Fill, SDI, Victoria, Australia	Bulk fill resin composite—BFRC	Diurethane dimethacrylate (3–20), triethylene glycol dimethacrylate (0.01–7), 2,2-bis[4-(2-methacryloxy)ethoxyphenyl]propane (15–18)	150931
Riva Self Cure, SDI, Victoria, Australia	Conventional glass ionomer cement—CGIC	Compartment 1: acrylic acid homopolymer (20–30), tartaric acid (10–15). Compartment 2: fluoro aluminosilicate glass (90–95).	B1510291F
Riva Light Cure, SDI, Victoria, Australia	Resin-modified glass ionomer cement—RMGIC	Compartment 1: 2-hydroxyethyl methacrylate (20–25), acrylic acid homopolymer (15–25), dimethacrylate cross-linker (10–25), tartaric acid (1–5). Compartment 2: glass powder (95–100).	J1508192EG
Riva Coat, SDI, Victoria, Australia	Coating agent	Acrylic monomer (100)	140339

^a^According to the material safety data sheet

### Preparation of the specimens

**Fig. 1:**
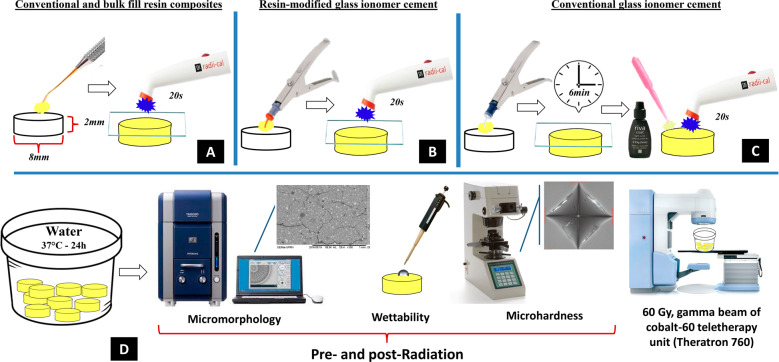
Schematic representation of the methods used in this study. Specimens of conventional and bulk-fill resin composites (**A**), conventional glass ionomer cement (**B**), and resin-modified glass ionomer cement (**C**) were produced according to manufacturers’ directions. Then, after 24 h of storage in water, micromorphology in Scanning Electron Microscopy, wettability through the sessile drop method, and Vickers microhardness were evaluated. Specimens were exposed to gamma radiation and the same surface parameters were analyzed (**D**)

A schematic representation of the methods is shown in Fig. 1. Forty disc-shaped specimens (*n* = 10) were produced (8 mm diameter × 2 mm thickness) according to the materials used in this study. Resin composites were inserted in a single increment into the mold and covered with a mylar strip and a 1-mm thick glass slide before photoactivation for 20 s using a light-emitting diode (Radii-Cal, SDI, Victoria, Australia—1200 mW/cm^2^, 440–480 nm). Glass ionomer cement encapsulated were prepared according to the manufacturer’s instructions. The plunger was placed on a hard surface and a mechanical mixer (Ultramat S, SDI, Victoria, Australia, 4600 rpm) was used to mix the capsules for 10 s. The capsule was then placed into the Riva applicator (SDI Limited, Bayswater, VIC, Australia). Then, a single portion of materials was inserted into the mold and covered with a mylar strip and a 1-mm thick glass slide. The resin-modified glass ionomer cement was photoactivated for 20 s. The self-cure glass ionomer cement was kept undisturbed for 6 min until the entire curing, and the coating agent (Riva Coast, SDI Limited, Victoria, Australia) was applied and photoactivated for 20 s. Specimens were stored in water at 37 °C for 24 h before further analysis.

### Baseline analyses

Micromorphology, wettability, and microhardness were evaluated in all specimens. Photomicrographs were obtained through Scanning Electron Microscopy (SEM) (TM-3000, Hitachi Tabletop, Tokyo, Japan) with 100X magnification on the center of each specimen to observe how the surfaces of the glass ionomers and resin composites behaved before and after exposition to ionizing radiation. The specimens did not need any surface treatment because an Environmental SEM was used.

Wettability was evaluated by measuring the contact angle between all materials’ surfaces and distilled water through the sessile drop method at room temperature [26] with a drop shape analysis system (Surftens 4.7 Software, Frankfurt, Germany). A drop of 10 µl of distilled water was dispensed on the center of each specimen and the contact angle was measured at three different locations for each specimen and the average value was reported.

Microhardness was measured using a digital microhardness tester (MV2000A, Pantec, São Paulo, SP, Brazil). Five indentations were made on the center of specimens randomly under 50 g load for 15 s. The diagonals of Vickers indentations were measured through the eyepiece of the optical microscope immediately after indentation. The means of the diagonals of each indentation were measured and the mean of five indentations for each surface was calculated for each specimen.

### Irradiation

During radiation therapy, oral cancer patients are exposed to a total radiation dose ranged from 50 to 70.4 Gy [27]. Cobalt-60 or linear accelerator units can be used as a source of radiation [28]. To simulate the clinical parameters and radiation doses used during radiotherapy, this in vitro study used a linear accelerator to apply the radiation dose of 60 Gy on forty disc-shaped specimens in a single session [29], with a gamma beam of cobalt-60 teletherapy unit (Theratron 760) simulating a radiotherapy procedure applied to patients with head and neck cancer. Radiation was performed in a hospital environment. The specimens were stored in distilled water and irradiated aiming at the homogeneity of irradiation.

### Post-radiation analyses

Forty-eight hours after irradiation, micromorphology, wettability, and microhardness were evaluated using the previously described protocol.

### Statistical analysis

Wettability (contact angles) and microhardness were evaluated using two-way ANOVA and Tukey posthoc tests (*p* < 0.05) since a parametric data distribution was obtained (Kolmogorov–Smirnov). The software ASSISTAT Beta (7.7 version) (Campina Grande, PB, Brazil) was used to perform statistic tests. Micromorphology was descriptively analyzed.

## Results

### Vickers microhardness

Two-way ANOVA revealed statistically significant differences among restorative dental materials (*p* < 0.001) and timepoints of analysis (*p* < 0.001). The interaction between “restorative dental material” versus “timepoint of analysis” was statistically significant (*p* < 0.001). Multiple comparisons among the groups are shown in Table 2. Bulk fill resin composite and resin-modified glass ionomer cement maintained statistically similar values before and after radiation. In contrast, conventional resin composite showed increased values, and conventional glass ionomer cement decreased values at post-radiation. At pre-radiation timepoint, resin composites showed statistically similar and lower values than glass ionomer cement. On the other hand, conventional resin composite showed statistically highest values at post-radiation timepoint, and conventional glass ionomer cement statistically the lowest values.

**Table 2:** Mean (standard deviation) of Vickers microhardness number according to restorative dental material and timepoint of analysis

Restorative dental material	Timepoint of analysis	
	Pre-radiation	Post-radiation
Conventional resin composite	61.97 (15.7)Bb	83.65 (16.0)Aa
Bulk fill resin composite	69.88 (3.9)Ab	68.95 (4.6)Abc
Conventional glass ionomer cement	76.26 (10.6)Aa	56.60 (4.0)Bc
Resin-modified glass ionomer cement	75.60 (18.5)Aa	73.95 (7.3)Aab

Different capital letters indicate statistically significant differences between time points within the same restorative dental material (*p* < 0.05). Different lowercase letters indicate statistically significant differences among restorative dental materials within the same time point (*p* < 0.05)

### Wettability

Two-way ANOVA revealed no statistically significant differences in time points of analysis (*p* = 0.3262). However, there were statistically significant differences among restorative dental materials (*p* = 0.0251) and the interaction between “restorative dental material” *versus* “timepoint of analysis” was statistically significant (*p* = 0.0194). Multiple comparisons among the groups are shown in Table 3. Conventional glass ionomer cement showed statistically lower values post-radiation than at pre-radiation timepoint, whilst other restorative dental materials maintained statistically similar values between different time points. At pre-radiation, resin composites showed similar values and statistically lower than those of glass ionomer cement. On the other hand, restorative dental materials showed statistically similar values post-radiation.

**Table 3:** Mean (standard deviation) of contact angles according to restorative dental material and timepoint of analysis

Restorative dental material	Timepoint of analysis	
	Pre-radiation	Post-radiation
Conventional resin composite	48.40 (10.5)Ab	55.20 (6.8)Aa
Bulk fill resin composite	50.10 (6.9)Ab	55.50 (6.1)Aa
Conventional glass ionomer cement	62.90 (6.2)Aa	55.28 (8.3)Ba
Resin-modified glass ionomer cement	55.10 (4.2)Aa	57.20 (7.9)Aa

Different capital letters indicate statistically significant differences between time points within the same restorative dental material (*p* < 0.05). Different lowercase letters indicate statistically significant differences among restorative dental materials within the same time point (*p* < 0.05)

### SEM analysis

**Fig. 2:**
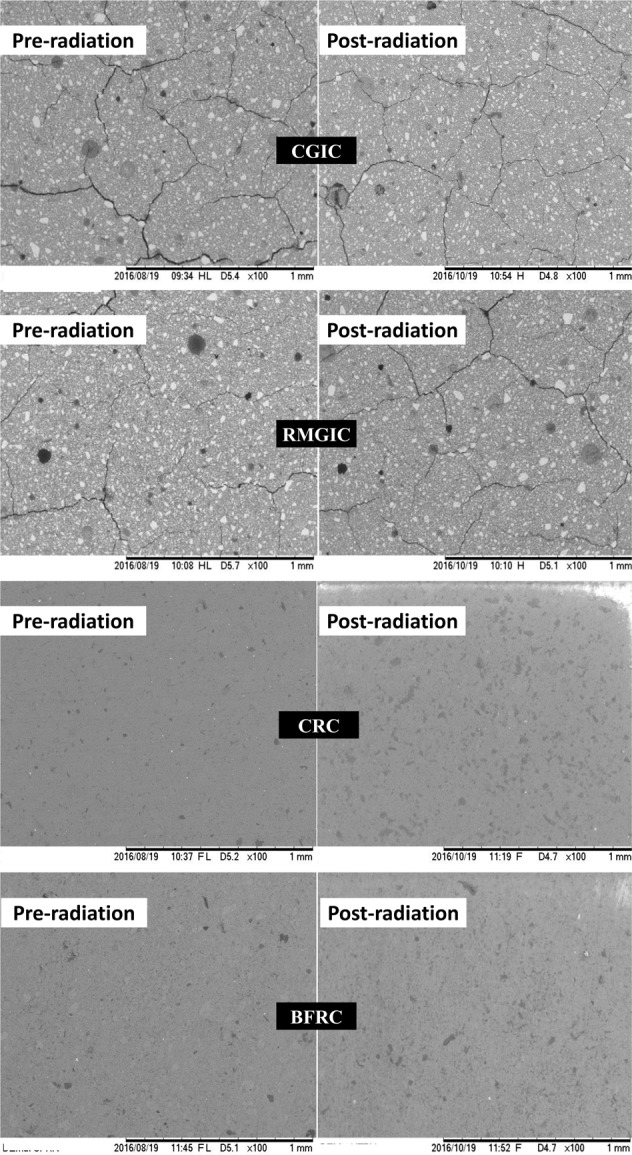
Images of the conventional glass ionomer cement (CGIC) Riva self cure, resin-modified glass ionomer cement (RMGIC) Riva light cure, conventional resin composite (CRC) Aura Enamel, and bulk-fill resin composite (BFRC) aura bulk fill with 100× magnification before (left) and after exposition to ionizing radiation (right)

Figure 2 presents images of the materials before and after ionizing radiation. In the CGIC, ionizing radiation removed the resin coating that was applied to the material surface. On the other hand, ionizing radiation did not alter the morphology of RMGIV, which showed similar visual characteristics at baseline and post-radiation. In the CRC, specimens after ionizing radiation presented more filler particles exposed on the material surface, which were covered by the organic matrix at baseline. Specimens of BFRC after ionizing radiation presented fewer large filler particles exposed and smaller filler particles exposed than at baseline.

## Discussion

The null hypothesis—that ionizing radiation would not alter the surface properties of materials—was rejected. In general, ionizing radiation promoted changes in the microhardness, wettability, and surface morphology of resin composites and glass ionomer cements tested.

In this study, a conventional and a resin-modified GIC, and a conventional and a bulk-fill resin composite were tested as they are the most commonly used dental materials to perform direct tooth restorations [11] and can be subjected to ionizing radiation in individuals undergoing head and neck radiotherapy. CGIC’s are produced by an acid-base reaction from a powder–liquid mixture [30]. Whilst the liquid contains mainly water and polyacrylic acid, the powder contains non silanized fluoro-alumino-silicate fillers (FASF) and other inorganic components such as strontium, phosphate, zinc, calcium, or sodium which react with [31]. RMGIC contains the same components as CGIC, but the FASF are silanized and the liquid includes methacrylate monomers, typically 2-hydroxyethyl methacrylate (HEMA), and camphorquinone as photoinitiator [32]. On the other hand, resin composites are composed of methacrylate monomers, photoinitiators, typically camphorquinone, and silanized filler particles [33].

When dental materials are subjected to ionizing radiation, they can interact with their surface and cause structural changes, which will occur distinctly depending on the surface chemical components of the material. The CGIC tested was the only one that presented microhardness decreasing after being exposed to ionizing radiation. Ionizing radiation likely caused the dissolution of resin coating applied on the CGIC’s surface and detachment of their non silanized FASF, exposing a softer material with decreased microhardness. Silane coupling agents are mainly organic silicides (X_3_SiY) where X may be chlorine, alkoxy or acetoxy groups and Y may be vinyl, epoxy, amino or mercapto groups. X groups convert to the alkoxy group via hydrolysis and make hydrogen bonding or covalent bonding with the alkoxy group present on the surface of inorganic filler particles while Y is reactive groups that bind with organic monomers and hence improve adhesion of interface [34]. Since FASF are not silanized and CGIC has not methacrylate monomers, probably, they were numerously detached from the material surface, which would have led to a microhardness decreasing. Conversely, as the RMGIC tested contains silanized FASF and methacrylate monomers, a more stable bond between monomers and fillers can be obtained [33] so that they were less detached, and post-radiation morphology microhardness was maintained.

Concerning CRC and BFRC tested, SEM images showed that a slight dissolution of materials’ surfaces occurred in consequence of exposition to ionizing radiation, which promoted the appearance of subsurface filler particles that were distributed below the resin-rich surface layer [35, 36]. As it was seen at baseline, the CRC Aura Enamel contains only microfillers of similar size, which were exposed after the dissolution of the resin matrix occurred after the specimens were subjected to ionizing radiation. On the other hand, the BFRC aura bulk fill presented filler particles with different sizes even at baseline. Ionizing radiation likely weakened the bond between larger fillers and resin matrix due to a larger peripherical area exposed than in smaller particles. In this way, larger particles would have been detached from the polymerized resin matrix, appearing smaller particles that were maintained entrapped into the polymerized resin matrix.

In summary, the hardness of restorative materials depends on the number of exposed filler particles [37]. Thus, the higher exposition of filler particles provided increased surface hardness values in the conventional resin composite tested. Also, a glass ionomer cement is softer than other restorative materials [38]. In this way, the resin coat remotion in the conventional glass ionomer cement post-radiation exposed a softer and more hydrophilic material with a decreased hardness and increased wettability. Also, conventional glass ionomer cement is softer and more hydrophilic than other restorative materials [39].

At the moment that ionizing radiation can cause a dissolution of the resin matrix, it can improve linking among polymerized chains after photoactivation through molecular excitation and continuous polymerization of the non-polymerized surface layer [25, 40]. Polymerized chains can form crosslinks through hydrogen bonds between OH or NH groups and ether or carbonyl groups, as well as among themselves, especially for hydroxyl-hydroxyl groups of monomers [41, 42]. In this way, resin composites containing a higher percentage of dimethacrylate monomers that present these groups, such as bisphenol A diglycidyl methacrylate (Bis-GMA), diurethane dimethacrylate (UDMA), and ethoxylated bisphenol A dimethacrylate (Bis-EMA). Since only the CRC showed increased microhardness at post-radiation, Aura Enamel probably contains an increased percentage of monomers that can form post-polymerization crosslinks than the BFRC aura bulk fill. It is reasonable to assume that the decreased microhardness of resin composites at post-radiation obtained previously [20], different from the present investigation, may be attributed to the lower percentage of crosslinker monomers in the materials tested. From a clinical point of view, it is supposed that Eura Enamel may provide increased clinical longevity of tooth restorations in individuals undergoing head and neck radiotherapy, once microhardness is related to the material’s strength [43].

Another important parameter that influences the longevity of resin composite restorations is how distilled water interacts with the material surface. High contact angle values imply lesser staining, biofilm accumulation, and pathogen adhesion/proliferation and, ultimately, lower the risk of caries in the restoration margins and progression [44]. Conversely, increased wettability (low contact angle values) of the restorative material can favor continuous penetration of water or oral solvents, chemical degradation, and pore formation [45]. The fact that CFIC presented lower contact angles after exposition to ionizing radiation indicates that the material became more wettable. As resin composites and resin-modified glass ionomer cement contain organic monomers, they can have maintained the wettability of these materials at post-radiation. In this way, one can presume that the use of this CGIC to restore decayed teeth of individuals undergoing head and neck radiotherapy would not guarantee clinical longevity for dental restoration.

In a general way, the CRC showed the best behavior for all properties tested, although BFRC and CGIC were not negatively affected by ionizing radiation. Since individuals undergoing head and neck radiotherapy are more prone to have caries in the restoration margins [46], the ideal material to restore their decayed teeth should present mechanical strength, resistance to erosion, and inhibit the formation of caries lesions. Thus, although glass ionomer cement can release fluoride which contributes to inhibiting the formation of caries lesion [46], further in vivo studies should be performed to evaluate the behavior of the materials tested in this study. In clinical conditions, dental materials and tooth restorations are exposed to radiotherapy in the presence of saliva. However, the specimens were stored in distilled water in this study following previous investigations [24, 29, 47, 48]. Clinical variables associated with individual biology or oral hygiene such as hyposalivation, caries appearance, and longevity of restorative materials were not evaluated and, therefore, need to be evaluated in further clinical trials.

## Conclusion

The conventional glass ionomer tested was the only material tested which was negatively affected by exposition to ionizing radiation. Exposition to ionizing radiation positively affected the microhardness of the conventional resin composite tested, while did not alter its wettability, and maintained microhardness and wettability of the resin-modified glass ionomer cement and the bulk-fill resin composite tested.
